# Effect of modified radical laparoscopic hysterectomy versus open radical hysterectomy on short-term clinical outcomes in early-stage cervical cancer: a single-center, prospective, randomized controlled trial

**DOI:** 10.1186/s12957-023-03044-3

**Published:** 2023-06-03

**Authors:** Xin Lv, Bo Ding, JingYun Xu, Yang Shen

**Affiliations:** 1 School of Medicine, Southeast University, Nanjing, China; 2 Department of Obstetrics and Gynaecology, Zhongda Hospital, School of Medicine, Southeast University, Nanjing, 210009 China; 3 Institute of Sports and Health, 211112, 99 Lize Road, Max Science Park, Building 3, 7th & 8th Floor, Nanjing, China

**Keywords:** Early-stage cervical cancer, Laparoscopic surgery, Open surgery, Radical hysterectomy, Endocutter

## Abstract

**Background:**

The long-term prognosis of minimally invasive surgery and open surgery for early cervical cancer is controversial. This study mainly discusses the feasibility and effectiveness of the endocutter in radical laparoscopic hysterectomy for early cervical cancer.

**Methods:**

A single-center, prospective, randomized controlled trial of modified radical laparoscopic hysterectomy on patients with FIGO stage IA1 (lymphovascular invasion), IA2, and IB1 cervical cancer, between January 2020 and July 2021. Patients were randomly assigned into laparoscopic radical hysterectomy (LRH) and open radical hysterectomy (ORH) groups. The ORH group used right-angle sealing forceps for vaginal stump closure, whereas the LRH group used endoscopic staplers. The primary outcomes included the evaluation of the patient’s perioperative indicators, as well as short- and long-term complications. Recurrence and overall survival were considered secondary outcomes.

**Results:**

As of July 2021, 17 patients were enrolled in the laparoscopic surgery group and 17 in the open surgery group. The hospitalization time of the laparoscopic group was significantly shorter than those of the open group (15 min vs. 9 min, *P* < 0.001). The vaginal stump closure time in the laparoscopic group was longer than that in the open surgery group, and the difference was statistically significant (*P* < 0.001). Post-operative catheter removal (*P* = 0.72), drainage tube removal time (*P* = 0.27), number of lymph node dissections (*P* = 0.72), and incidence of intraoperative and post-operative complications between the two groups (*P* > 0.05). The median blood loss in the laparoscopic group was 278 ml, and it was 350 ml in the laparotomy group. The intraoperative blood transfusion rate was lower in the laparoscopic group; however, these differences did not reach statistical significance (*P* = 0.175). Vaginal margin pathology and peritoneal lavage cytology were negative, and all the patient’s vaginal stumps healed without infection. The median follow-up time of the laparoscopic group was 20.5 months, and it was 22 months for the open surgery group. There was no recurrence in all patients during the follow-up period.

**Conclusions:**

Modified LRH with endocutter closure of the vaginal stump is an effective approach and not inferior to ORH in treating patients with early-stage cervical cancer.

**Trial registration:**

ChiCTR2000030160, date of registration February 26, 2020 (https://www.chictr.org.cn/showprojen.aspx?proj=49809).

## Background

Cervical cancer incidence and mortality rates rank fourth among women’s cancer globally. The annual cervical cancer occurrence in 2020 was estimated to be more than 600,000 new cases, with a corresponding death toll of 340,000 patients [1]. Radical hysterectomy is the standard treatment recommended for early-stage cervical cancer [2]; however, the average recurrence rate is about 5.6% [3]. Previous guidelines [4] recommended that radical hysterectomy can be performed through open or minimally invasive surgical (MIS) techniques (including laparoscopy or robotics). Most previous studies have shown that the prognosis of patients with minimally invasive surgery for early-stage cervical cancer is not inferior to that of patients who undergo open surgery/laparotomy [5–7]. However, a randomized trial in 2018 showed that the incidence of disease-free survival (DFS) and overall survival (OS) in the MIS group were lower than that in the open surgery group [8]. Another retrospective study after the latter study also concluded that OS was shorter in the MIS group than in the laparotomy group [9]. Following these studies, questions about the safety of MIS arose, and further research identified intra-corporeal open colpotomy and the use of uterine manipulators as risk factors affecting the prognosis of cervical cancer patients with MIS [8, 10].

To this end, we used an observational, randomized controlled trial design to compare the clinical outcomes of modified laparoscopic radical hysterectomy and open radical hysterectomy. In addition, the study aimed to investigate the feasibility and safety of using an endoscopic stapler device to treat patients with **e**arly-stage cervical cancer while adhering to the principle of complete resection.

## Materials and methods

### Trial design

**Fig. 1:**
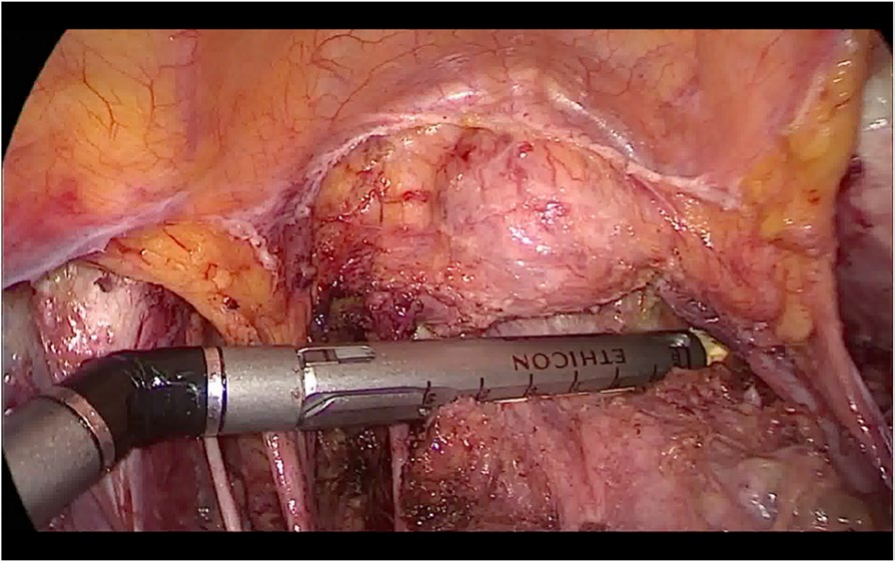
Laparoscopic vaginal stump closure using the endocutter

This study was a single-center, phase 2, randomized controlled trial registered under Chinese clinical trial number ChiCTR2000030160. Data was gathered from patients with FIGO (2018) stage IA1 (lymphovascular invasion), IA2, and IB1 cervical cancer at Zhongda Hospital of Southeast University in Nanjing, China, between March 2020 and July 2021. The institutional review board evaluated and approved relevant medical ethics issues (ethical approval number 2020ZDSYLL106-Y01). The study design flowchart is shown in Fig. 1.

### Sample size

The sample size was calculated according to the method specified in the previous study [11]. The 4.5-year DFS rate of patients undergoing radical hysterectomy for abdominal cervical cancer is estimated to be 90%. The selected non-inferiority margin is 15.0%. If the difference in disease-free survival rate is less than 15.0%, the two methods will be considered equivalent. The sample size required to evaluate the 4.5-year disease-free survival difference level was 70, the statistical power was assumed to be 80%, and the two-tailed test was 5% significant.

### Trial conduct and oversight

Patients who met the following criteria were included in the study: (1) FIGO2018 stage IA1 (with vascular tumor thrombus), IA2, IB1; (2) the largest tumor diameter < 2 cm (MRI evaluation or pathological evaluation after conization < 2 cm); (3) histological types: squamous cell carcinoma, adenocarcinoma, and adenosquamous carcinoma, except endometrioid adenocarcinoma; (4) patients with appropriate bone marrow hematopoietic function and renal function; (5) liver function: ((1) white blood cell count > 3.0 × 10^9^cells/L; (2) platelet count > 100 × 10^9^/L; (3) creatinine < 180 µmol/L; (4) bilirubin < 1.5 times normal, aspartate aminotransferase/alanine aminotransferase < 3 times normal); (6) Eastern Cooperative Oncology Group (ECOG) performance-status score of 0 or 1; (7) patients with surgical indications; (8) BMI < 35; (9) age range 18 to 70 years old; (10) patients who signed the informed consent.

Patients with the following conditions were excluded: (1) mental illness; (2) heart, liver, or kidney dysfunction; (3) bladder dysfunction before the operation or patients with other serious complications who could not bear the risk of surgery; (4) patients who had received radiotherapy or chemotherapy; (5) pregnant patients; (6) lost to follow-up; (7) tumor diameter ≥ 2 cm; imaging evaluation showing that the cervical tumor invades the outer 1/3 of the stroma or involves the lower uterine segment; (8) patients with FIGO stage IB2 and above; (9) patients whose medical compliance and geographic location could not guarantee sufficient follow-up; (10) patients with surgical contraindications; (11) BMI ≥ 35.

After signing a written consent, patients were randomly assigned by a network-based computer randomization program into the laparoscopy group and the laparotomy group at a ratio of 1:1. Masking was impossible because of the nature of the treatment.

All patients underwent extensive hysterectomy and pelvic lymph node dissection (with or without bilateral adnexectomy). The primary study objective was to compare the clinical outcomes between the laparoscopic group that used the endocutter stapler for vaginal stump closure and the laparotomy group that used right-angled forceps to close the vaginal stump. The primary outcomes of interest included the patients’ perioperative observation indicators and short- and long-term period complications. Recurrence and overall survival were considered secondary outcomes. The primary endpoint is 4.5 years of post-operative DFS (the time interval from the surgery date to the first recurrence).

### Surgical procedure

Following the induction of general anesthesia, the patient was placed in the lithotomy position, then disinfected and draped before access into the abdominal cavity was obtained.

The laparoscopic group had four surgical incisions on the patient’s abdominal wall: (1) a 10-mm camera port in the umbilicus, (2) a 5-mm port at the McBurney’s point on the right side, (3) an identical 12-mm working port on the left lateral lower quadrant, and (4) a 5-mm port parallel to the left mid-clavicular line 2 cm laterally at the level of the umbilicus. Abdominal pressure was set to 12 mmHg. The patients were adjusted to approximately 30° of Trendelenburg position. After entering the abdominal cavity, we carefully explored the pelvic and abdominal cavities to rule out peritoneal dissemination or distant metastasis. We adopted the no-lifting technique described in earlier investigations [12] instead of using a uterine manipulator.

Subsequently, pelvic lymphadenectomy was performed, and para-aortic lymph node dissection was performed where necessary. Pelvic lymph nodes were resected en bloc according to the order of common iliac → external iliac → deep inguinal → obturator → internal iliac vessels. After dissecting one side of the lymph nodes, it was bagged and sealed in time. The wound was repeatedly rinsed with sterile water for injection, and the contralateral lymph nodes were treated similarly.

Following the radical hysterectomy, the Ethicon Johnson & Johnson Echelon Flex Powered Plus Long Articulating Endoscopic Linear Cutter (Endocutter) with two rows of triple staggered titanium staples were inserted through the 12 mm trocar port. The angle between the jaws of the endocutter and the vagina’s longitudinal axis was adjusted to 90°, and then about 3 cm of the upper part of the vagina was closed after ensuring there was no abnormal tissue in the jaws except for the vaginal tissue. After the endocutter is fired, the device’s staples are released, and the vaginal stumps are sewn together on both sides while simultaneously cutting the vaginal wall (Fig. 1). The vaginal stump and row of staples were then resected, and the pneumoperitoneum was closed. After the uterine specimen was extracted through the vagina, the pelvic and abdominal cavities were lavaged with water for injection, and the washing liquid was sent for examination. The vaginal stump was then sutured with a 2/0 barbed suture, and the uterus specimen and vaginal stump were submitted for a histopathology examination.

In the ORH group, the patient was placed in the lithotomy position following induction of general anesthesia. A mid-line incision of 20 cm was made on the lower abdomen, and the rest of the procedure was carried out as stated in a previous detailed report [13]. Right-angle forceps were used to circularly excise the upper segment of the vagina at about 3 cm, and the pelvic and abdominal cavities were lavaged before the vaginal stump was sutured, and the washing fluid was submitted for a histopathology examination.

### Observation indicators

The patient’s general clinical and pathological information, such as age, body mass index, ECOG score, tumor grade, and pathological type, were recorded. Perioperative indexes include operation time (minutes), hospital stay (days), intraoperative complications (bleeding, major organ damage), intraoperative blood transfusion, lymphatic dissection, and the time to removal of the urinary catheter and drainage tube as well as the time spent dealing with the vaginal stump during the operation was recorded. Patients had regular follow-ups of their clinical condition, short-term post-operative indexes, post-operative complications, and pelvic lavage fluid cytopathology results were reviewed. Intraoperative complications, including bowel, bladder, ureter, nerve, or vascular injury, were considered short-term post-operative indexes. Early post-operative complications refer to vaginal stump infection and dehiscence within four weeks after surgery; long-term post-operative complications refer to incisional hernia and vaginal stump recurrence after 6 months.

### Statistical methods

The SPSS 22.0 software was used for the statistical analysis. Continuous data were analyzed using a *t*-test, and the categorical data were analyzed using the *χ*^2^ test. The Yates correction test was applied if the conditions were not met. A two-sided *P*-value < 0.05 indicated statistical significance. Descriptive statistical analysis was used for treatment-related adverse events; disease-free survival and median survival time were estimated using the Kaplan–Meier method. Disease-free survival was calculated from the surgery date to recurrence or the last follow-up time. The survival time was calculated from the time of diagnosis to the end of the follow-up period or time of death following diagnosis.

## Results

**Fig. 2:**
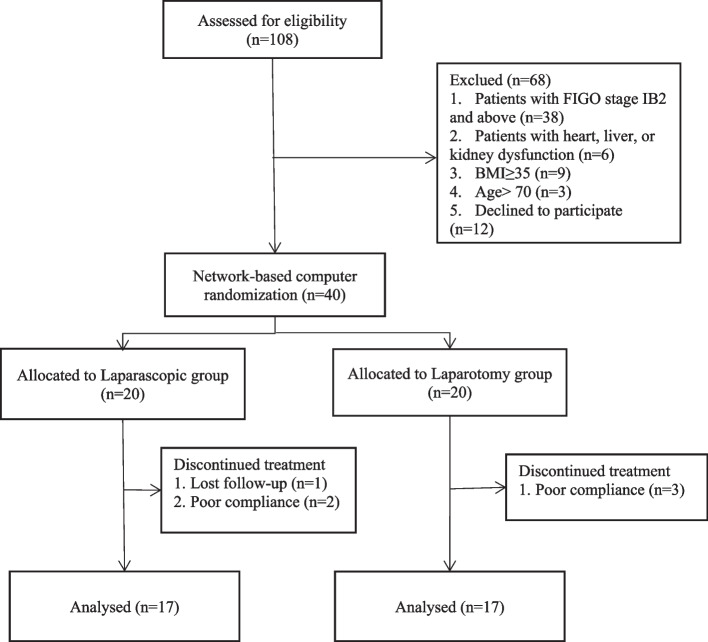
Study design flowchart

One hundred eight patients were recruited for surgical treatment of early-stage cervical cancer. Forty met the eligibility criteria; 20 underwent modified minimally invasive hysterectomy, and 20 underwent open radical hysterectomy. Six patients were excluded after allocation to the two groups, and of these, five patients were excluded because they did not comply with post-operative adjuvant treatment, and 1 patient was lost to follow-up, as shown in Fig. 2.

Table 1 displays the patients’ general clinical information and pathological traits. There was no statistical difference between the average age, body mass index, and pathological type of laparoscopic and laparotomy groups. The baseline characteristics of the two groups were identical, and no patients undergoing LRH converted to ORH.

**Table 1:** General clinical data and pathological features of the patients at baseline

Characteristics	Laparoscopic group (*N* = 17)	Laparotomy group (*N* = 17)	*P*-value
Age (years)	48.29 ± 3.18	50.48 ± 2.80	0.28
BMI (kg/m^2^)	24.12 ± 1.16	24.70 ± 0.78	0.89
ECOG score	0–1	0–1	
Pathological type			0.98
Squamous cell carcinoma	13	12	
Adenocarcinoma, adenosquamous carcinoma	4	5	
FIGO stage			1.00
IA	8	8	
IB1	9	9	
Tumor grading			1.00
G1	0	0	
G2	13	12	
G3	4	5	

FIGO stage: an International Federation of Gynecology and Obstetrics 2018 staging

*BMI *body mass index, *ECOG score *Eastern Cooperative Oncology Group

Values are presented as mean ± standard deviation and absolute number

The perioperative and follow-up indexes of the two groups are shown in Table 2. The laparotomy group’s mean vaginal stump closure time was relatively shorter (9.56 min vs. 15.31 min, *P* < 0.05). The average operation time was 202 and 216 min in the laparoscopic and laparotomy groups, respectively. There was no significant difference in the average length of hospital stay and the median time of urinary catheter and meantime of drain tube removal, which was 14 days vs. 16 days, 14 days vs. 10 days, and 7.5 days vs. 8.3 days, respectively. The two groups had no statistical difference in the number of dissected lymph nodes. The median blood loss in the laparoscopic group (278 ml) was 3/4 of the median blood loss (400 ml) in the laparotomy group, and the intraoperative blood transfusion rate was lower in the laparoscopic group; however, these differences did not reach statistical significance (*P* > 0.05).

**Table 2:** Perioperative period and follow-up data and indicators

Parameters	Laparoscopic group (*N* = 17)	Laparotomy group (*N* = 17)	*P*-value
Operation time (minutes)	202.19 ± 7.17	216.15 ± 6.94	0.18
Length of hospital stay (days)	14.63 ± 0.85	16.77 ± 0.85	0.09
Intraoperative complications			
Blood loss (ml)	278 (200,400)	350 (250,450)	0.04
Intestinal injury	0	0	
Ureteral injury	0	0	
Bladder injury	0	0	
Intraoperative blood transfusion (ml)			0.175
Yes	1	5	
No	16	12	
Number of lymph nodes removed	25 (16.5,33)	28 (20,34)	0.49
Urethral catheter removal time (days)	14 (8,14.5)	10 (8,13)	0.72
Drain removal time (days)	7.50 ± 0.50	8.38 ± 0.63	0.27
Vaginal stump closure time (minutes)	15.31 ± 0.33	9.56 ± 0.53	0.0001
Post-operative complications			
^ a^Vaginal stump complications	0	0	1.00
^ b^Lymphatic complications	0	2	0.48
^ c^Urologic complications	4	2	0.66
Positive cytology of pelvic lavage fluid	0	0	1.00
Positive pathological of vaginal margins	0	0	1.00
The median time of follow-up (months)	20.5 (18,36)	22 (18,36)	
Cancer recurrence	0	0	1.00
Post-operative deaths	0	0	1.00

Values are presented as mean ± standard deviation, absolute number, median (interquartile range)

^a^Vaginal stump complications include vaginal stump infection, dehiscence, and poor healing

^b^Lymphatic complications include lymphocele, abscess, edema, and lymphatic fistula

^c^Urological complications include urinary tract infections and urinary retention

No lymphadenectomy-related complications were reported in the laparoscopic group; however, in the laparotomy group, one patient developed a lymphatic cyst on the 13th post-operative day and another patient at 1 month post-operative. Post-operative urinary system complications occurred in 6 cases. Among these, 3 cases of urinary retention and 1 cystitis occurred in the laparoscopic group, while urinary tract infection occurred in the laparotomy group. There was no statistical difference in post-operative adjuvant radiotherapy between the two groups. The vaginal stumps of all patients in the two groups healed well without any complications. All patients had negative pathological examination results of vaginal surgical margins and negative peritoneal lavage cytology examination. During the follow-up period, the vaginal incision site of patients in the laparoscopic group had healed well, and no vaginal incision dehiscence or infection occurred. The median follow-up time of the laparoscopic and laparotomy groups was 20.5 months and 22 months, respectively. All patients survived without disease recurrence.

## Discussion

The use of LRH for cervical cancer was first reported in 1992 [14]. Laparoscopic surgery has distinct advantages over open surgery, including less trauma, pain, and bleeding and reduced infection rates [15, 16]. Due to these benefits, laparoscopic surgery for cervical cancer has quickly gained popularity among medical professionals and patients. A 2018 LACC trial showed that the open surgery group’s progression-free and overall survival rates were significantly higher than those of the laparoscopic group. Based on the LACC study, the updated National Comprehensive Cancer Network Guidelines (3rd edition) in 2019 only recommends open surgery for patients with early cervical cancer [17]. However, some studies have questioned the results of the LACC study and sighted a significant flaw due to missing follow-up data, which may affect the results. A 2022 study on minimally invasive surgery for early cervical cancer showed that [18] the shift from minimally invasive to open radical hysterectomy does not affect the 90-day surgery-related morbidity of patients. Some studies have pointed out that [19] there was no significant difference in the incidence of intraoperative and post-operative complications between radical laparoscopic hysterectomy and open radical hysterectomy. In addition, recent systematic review and meta-analysis also showed that laparoscopic-assisted vaginal radical hysterectomy had no significant effect on progression-free survival and overall survival in patients with early cervical cancer [20]. It can be seen that the clinical controversy about laparoscopic surgery and open surgery mainly focuses on the post-operative prognosis.

The main principles of tumor surgery include the prevention of possible tumor extravasation; however, cervical cancer itself possesses the characteristic of spreading outward and toward the vagina, which can complicate surgery. The findings of the 2018 LACC trial [8] shed light on the safety concerns surrounding MIS. The trial reported that several steps during minimally invasive surgery (MIS) may increase the risk of tumor extravasation. Uterine manipulators used in MIS may increase tumor spillage tendency and destroy tumor integrity, promoting dissemination [21]. A study in the USA in 2019 found that all 26 patients that did not use a uterine lifter during MIS for early-stage cervical cancer patients had no recurrence; still, the risk of recurrence in the MIS group was significantly higher than that of the open surgery group [21]. Likewise, the 2020 SUCCOR study pointed out [22] that the recurrence rate of early cervical cancer among patients who used uterine manipulators in laparoscopic surgery was 2.76 times that of patients who had open surgery. The study also noted no difference in the recurrence rates between patients who did not use uterine manipulators during laparoscopic surgery and those who had open surgery.

Moreover, it is believed that cancer cells can detach from the tumor surface during surgery for cervical malignancies and contaminate the carbon dioxide-filled peritoneal cavity. In a study by Kong et al. [23], the disease recurrence rate was higher in the internal colpotomy group compared with the vaginal colpotomy group (16.3% vs. 5.1%, *P* = 0.057). They suggested that, in contrast to vaginal colpotomy, internal colpotomy performed under pneumoperitoneum pressure may be associated with an increased risk of intraperitoneal tumor extravasation facilitated by circulating carbon dioxide. Based on these findings, the European Society of Gynecological Endoscopy recommended that every effort be made to avoid tumor extravasation and peritoneal contamination during radical hysterectomy [24].

Furthermore, during intraabdominal vaginotomy, the last step during LRH, intra-abdominal exposure to the tumor while separating the vagina could easily cause abdominal and pelvic dissemination and increase the risk of tumor spillage. In order to avoid the direct exposure of tumor tissue to the abdominal cavity, open surgery often uses preoperative vaginal fornix clamping, but laparoscopic radical hysterectomy cannot routinely replicate this step. It can be achieved by creating vaginal cuffs or closing vaginal cuffs by suture or permanent tie. Yuan et al. [25] reported a transvaginal closure method in which the upper vagina was ligated before vaginotomy; this method could effectively prevent tumor spillage. Other techniques have been reported, such as clamping the vagina with a clip [26] or vaginal cuff closure with sutures [27] and surgical staplers. However, these vaginal closure methods still have risks, such as incomplete vaginal closure and increased tumor contamination.

Therefore, we modified our laparoscopic surgery as follows: (1) instead of using a uterine manipulator, we used transuterine suspension sutures, which ensured that the angle and position of the uterus could be adjusted for complete exposure of the surgical field of view without squeezing or destroying the tumor. (2) In this trial, cutting and closing the vagina with the endocutter instead of the traditional way of closure allowed for a more straightforward procedure, with enough room for complete vaginal wall and cervical lesion resection. At the same time, it ensured the safety of the vaginal resection margin and reduced the spillage of intraperitoneal tumors. Compared with the previous vaginal suture methods, the endocutter closes the vaginal vault more evenly and tightly, and the closure is superior. (3) The complete resection (tumor-free) principle was strictly implemented in this trial. Previous studies have identified an association between positive vaginal excision margins and an increased risk of early-stage cervical cancer recurrence along residual vaginal tissue [11, 28, 29]. To further ensure the safety of vaginal resection margins in the experiment, the additional resected vaginal margins with staples were sent for secondary pathological examination. Also, the cytology examination of the peritoneal lavage contents obtained after hysterectomy in this study did not show any tumor cells, and all of the vaginal resection margins were negative, which reduced the risk of recurrence.

The vaginal stump treatment time in the laparotomy group was significantly shorter than in the laparoscopic group. This may be related to the limitation of the laparoscopic technique itself; time is saved when using the right-angle sealing forceps compared with the endocutter device. It takes more time to follow the principle of complete resection when working under a limited field of view, and it also requires more time to extract the specimen from the vagina than direct extraction during laparotomy. However, this study found no significant difference in the total operation time between the two groups, suggesting that time spent before the treatment of the vaginal stump during the initial steps of the laparoscopic procedure can be optimized. Steps such as skin incision are relatively faster than in laparotomy; laparoscopic pelvic lymph node dissection time is shorter than open radical hysterectomy, mainly because laparoscopy has a high resolution and provides more accurate anatomy exposure, and more energy devices such as bipolar coagulation used for hemostasis are less time-consuming.

From a subjective point of view, the study guaranteed the quality of the operations. The trial facility is a quality control pilot unit of the National Cancer Center and is one of China’s top 50 tertiary hospitals. The patients used in the trial were strictly screened, and the surgical staff involved have all passed strict certification procedures; they have rich experience, and all operations were completed independently. This study also has the benefit of using a prospective randomized controlled design to assess short-term clinical outcomes and the efficacy of a modified minimally invasive radical hysterectomy. These findings will help plan and design subsequent studies on the safety and efficacy of early-stage cervical cancer surgical treatment.

## Limitations

This study has shortcomings; when a negative washing cytology result is obtained, although it is technically tumor-free, it does not mean that the prognosis of the tumor is good, and there may be unexpected metastatic disease. At this time, it should be interpreted carefully in conjunction with the pathological results. In addition, using the endocutter can increase potential surgical costs. Although the sample size of this report is still small and the follow-up time is short, further long-term follow-up data will be supplemented in the future as this trial is still ongoing at the time of this report. Nevertheless, the findings strengthen our confidence in the continued use of MIS for patients with early-stage cervical cancer.

## Conclusion

In this study, neither recurrence nor mortality occurred in either of the patient groups during the follow-up period. It demonstrates that using the endocutter to close the vaginal stump during laparoscopy for radical hysterectomy is effective for treating patients with early-stage cervical cancer. This technique deserves to be advocated for because the clinical curative outcome is comparable to laparotomy.

## Data Availability

The datasets generated during and/or analyzed during the current study are available from the corresponding author upon reasonable request.

## References

[1] Sung H, Ferlay J, Siegel RL (2021). Global cancer statistics 2020: GLOBOCAN estimates of incidence and mortality worldwide for 36 cancers in 185 countries[J]. CA Cancer J Clin.

[2] Bu-Rustum NR, Yashar-CM, Bean S, et al.NCCN guidelines insights: cervical cancer, version1.2022 [EB/OL].(2021–10–26)[2022–05–01]. https://www.nccn.org/quidelines/quidelines-detail?category=1&id=1426. Accessed 1 Feb 2023.

[3] Nitecki R, Ramirez PT, Frumovitz M (2020). Survival after minimally invasive vs open radical hysterectomy for early-stage cervical cancer: a systematic review and meta-analysis. JAMA Oncol.

[4] Koh WJ, Abu-Rustum NR, Bean S, et al. Cervical cancer, version 3.2019, NCCN clinical practice guidelines in oncology. J Natl Compr Cancer Netw. 2019; 17(1):64–84. 10.6004/jnccn.2019.0001 30659131

[5] Corrado G, Vizza E, Legge F (2018). Comparison of different surgical approaches for stage IB1 cervical cancer patients: a multi-institution study and a review of the literature. Int J Gynecol Cancer.

[6] Diver E, Hinchcliff E, Gockley A (2017). Minimally invasive radical hysterectomy for cervical cancer is associated with reduced morbidity and similar survival outcomes compared with laparotomy [J]. J Minim Invasive Gynecol.

[7] Gallotta V, Conte C, Federico A (2018). Robotic versus laparoscopic radical hysterectomy in early cervical cancer: a case matched control study. Eur J Surg Oncol.

[8] Ramirez PT, Frumovitz M, Pareja R (2018). Minimally invasive versus abdominal radical hysterectomy for cervical cancer [J]. N Engl J Med.

[9] Melamed A, Margul DJ, Chen L (2018). Survival after minimally invasive radical hysterectomy for early-stage cervical cancer. N Engl J Med.

[10] Lago V, Tiermes M, Padilla-Iserte P, Matute L, Gurrea M, Domingo S (2021). Protective maneuver to avoid tumor spillage during laparoscopic radical hysterectomy: vaginal cuff closure. J Minim Invasive Gynecol.

[11] Park SJ, Kong TW, Kim T, Lee M, Choi CH, Shim SH, Yim GW, Lee S, Lee EJ, Lim MC, Chang SJ, Lee SJ, Lee SH, Song T, Lee YY, Kim HS, Nam EJ (2022). Safety and efficacy study of laparoscopic or robotic radical surgery using an endoscopic stapler for inhibiting tumor spillage of cervical malignant neoplasms evaluating survival (SOLUTION): a multi-center, open-label, single-arm, phase II trial protocol. BMC Cancer.

[12] Ding B, Guan X, Duan K, Shen Y. Laparoscopic radical hysterectomy with enclosed colpotomy without the use of uterine manipulator for early-stage cervical cancer. J Minim Access Surg. 2021;17(4):570-572. 10.4103/jmas.JMAS_146_20 PMC8486072 34558435

[13] Abu-Rustum NR, Hoskins WJ (2001). Radical abdominal hysterectomy. Surg Clin North Am.

[14] Nezhat CR, Burrell MO, Nezhat FR (1992). Laparoscopic radical hysterectomy with paraaortic and pelvic node dissection. Am J Obstet Gynecol.

[15] Wang Y, Deng L, Xu H (2015). Laparoscopy versus laparotomy for the management of early stage cervical cancer. BMC Cancer.

[16] Janda M, Gebski V, Davies LC (2017). Effect of total laparoscopic hysterectomy vs total abdominal hysterectomy on disease-free survival among women with stage I endometrial cancer: a randomized clinical trial. JAMA.

[17] Koh WJ, Abu-Rustum NR, Bean S, et al. Cervical Cancer, Version 3.2019, NCCN Clinical Practice Guidelines in Oncology. J Natl Compr Canc Netw. 2019;17(1):64–84. 10.6004/jnccn.2019.0001 30659131

[18] Bogani G, Donato VD, Scambia G (2022). Practice patterns and 90-day treatment-related morbidity in early-stage cervical cancer. Gynecol Oncol.

[19] Pecorino B, D’Agate MG, Scibilia G (2022). Evaluation of surgical outcomes of abdominal radical hysterectomy and total laparoscopic radical hysterectomy for cervical cancer: a retrospective analysis of data collected before the LACC trial. Int J Environ Res Public Health.

[20] Ronsini C, Köhler C, De Franciscis P (2022). Laparo-assisted vaginal radical hysterectomy as a safe option for minimal invasive surgery in early stage cervical cancer: a systematic review and meta-analysis. Gynecol Oncol.

[21] Li R-Z, Sun L-F, Li R, Wang H-J (2023). Survival after minimally invasive radical hysterectomy without using uterine manipulator for early-stage cervical cancer: a systematic review and meta-analysis. BJOG.

[22] Chiva L, Zanagnolo V, Querleu D, Martin-Calvo N, Arévalo-Serrano J, Căpîlna ME (2020). SUCCOR study: an international European cohort observational study comparing minimally invasive surgery versus open abdominal radical hysterectomy in patients with stage IB1 cervical cancer. Int J Gynecol Cancer.

[23] Kong TW, Chang SJ, Piao X (2016). Patterns of recurrence and survival after abdominal versus laparoscopic/robotic radical hysterectomy in patients with early cervical cancer. J Obstet Gynaecol Res.

[24] Klapdor R, Hertel H, Hillemanns P (2019). Peritoneal contamination with ICG-stained cervical secretion as surrogate for potential cervical cancer tumor cell dissemination: a proof-of-principle study for laparoscopic hysterectomy. Acta Obstet Gynecol Scand.

[25] Yuan P, Liu Z, Qi J, et al. Laparoscopic radical hysterectomy with enclosed colpotomy and without the use of uterine manipulator for early-stage cervical cancer. J Minim Invasive Gynecol. 2019;26(6):1193–1198. 10.1016/j.jmig.2019.01.016 30802608

[26] Sekiyama K, Ando Y, Taga A (2020). Laparoscopic technique for step-by-step nerve-sparing Okabayashi radical hysterectomy. Int J Gynecol Cancer.

[27] Kalkan Ü, Bakay K (2022). A multimodal concept for vaginal cuff closure by modification of the Bakay technique in total laparoscopic hysterectomy: a randomized clinical study. BMC Womens Health.

[28] Von Mehren M, Kane JM, Bui MM, et al. NCCN guidelines insights: soft tissue sarcoma, version 1.2021: featured updates to the NCCN guidelines [J]. Journal of the National Comprehensive Cancer Network, 2020;18(12):1604-1612. 10.6004/jnccn.2020.0058 33285515

[29] Uppal S, Gehrig PA, Peng K (2020). Recurrence rates in patients with cervical cancer treated with abdominal versus minimally invasive radical hysterectomy: a multi-institutional retrospective review study. J Clin Oncol.

